# Measuring demand and supply of community care services for older people healthy ageing in rural Zhejiang Province

**DOI:** 10.1186/s12877-022-02906-x

**Published:** 2022-04-06

**Authors:** Wusi Zhou, Biya Jiang, Liujun Yu, Weidong Dai

**Affiliations:** 1 School of Public Administration, Hangzhou Normal University, Hangzhou, 311121 China; 2 School of Public Administration, Zhejiang University of Finance and Economics, 310018 Hangzhou, China; 3 China Institute of Regulation Research, ZUFE, 310018 Hangzhou, China

**Keywords:** Rural ageing, Family care, Older people, Community prosperity

## Abstract

**Background:**

A consequence of demographic trends and economic prosperous is the increasing diversity in needs for care services. However, the traditional family support for older people has been largely supplanted by the wider provision of community care services. This study aims to investigate the current status of demand and supply in community care services across different villages of Zhejiang province and assess service effectiveness for healthy ageing.

**Methods:**

A questionnaire survey was carried out towards 207 rural villages across 9 cities in Zhejiang province. One hundred eighty-six valid responses were received, representing a response rate of around 89.9%. Descriptive statistics were employed to identify older villagers’ care needs and available community services. Comparative analysis examined the balance between the demand and supply of community care services. Correlation analysis were applied to determine key factors that impacted the supply of social services in rural communities.

**Results:**

The research found that rural older residents normally lived with their children or spouse with limited literacy and income. On average the categories of community care services is substantially small in comparison with the increasingly diverse demands of older people in rural areas. There was an obvious mismatch between service demand and service supply in rural communities, which often caused the waste of public resources. Moving forward, the uppermost priority is given to infrastructure construction service and daily life service, while little attention is paid to mental health service and specialized nursing service.

**Conclusion:**

There needs to be an improvement in the socio-economic capacity of rural communities and in the diversity of social care services. Policies and strategies are also needed to encourage private sectors’ involvement in providing care services for rural older people. Local government should have a clear vision of the potential demands for community care services, practical guidelines will be useful in guaranteeing better service quality.

## Introduction

Demographic transition is clearly pointing to an ageing population. Based on the World Population Ageing [1], 703 million people were aged 65 and over across the world in 2019, the number is expected to double to 1.5 billion in 2050. That is, in 30 years from now, around 1 in 6 persons is projected to be at least 65 years old [2]. This highlights an increasing need for the improvement of health and well-being as well as some challenges to the sustainability of the pension system. There have been also very rapid demographic shifts in China. The Seventh National Population Census of China confirmed that, there were 264 million older people aged 60 and over by the end of 2020, taking up 18.7% of the national population [3]. This number is estimated to reach 34.1% (around 483 million) in 2050 by the Annual Report on China’s Health Industry Development 2018 [4]. At that time, China will enter into a super-aged society, with the remarkable decline in mortality and fertility [5].

Since the majority of older people live in rural China, villages are growing older more quickly than cities [6]. Until 2020, the number of older population aged 60+ and 65+ in rural areas accounted for 23.8 and 17.7% respectively, around 8.0 and 6.6% larger than that in urban areas [3]. Between 2018 and 2019, there was an obvious decrease of rural population from 39.4 to 40.4% [7], while the share of rural older population was seen a slight increase to 20.8% from 20.4% [8]. This signifies a steady growth of the dependence ratio. Due to the decline in fertility and the rise in longevity, Chinese families are turning to be smaller and older [9]. According to the China Population and Employment Statistics Yearbook 2020, the average old-age dependency ratio in rural regions was 22.3% in comparison with 14.5% in urban cities in 2019. By 2030, the rural old-age dependency ratio is projected to rise sharply up to 79.9%, double the urban ratio of 36.9% [10]. In addition, following the process of modernisation and urbanisation, labor flow from rural to urban areas has been mounting perceptibly [11], leading to a steady rise in the number of left-behind older people. The Sixth National Population Census of China reported that there were 30.77% emply-nest families in rural areas [12]. This trend continues to grow, the percentage of left-behind older people is predicted to reach 26.1% in rural areas by 2050, which will be nearly three times over the number in urban [13]. There has been a noticeable gap between old-age dependency and empty-nest households, which brought an incredible number of older people seeking community care services for the purpose of successful ageing in place [14].

Community care services refer to a range of health and social care services (e.g. daily care, recreation activities) that are provided by local communities for older people to live independently in their own home and familiar neighborhood [15, 16]. Rural economic development and improved quality of life are creating diversity in needs of older people for community care services [17]. Notably, there is the conceptual distinction between needs and demands when formulating welfare policies and providing public services [18]. Needs are normally defined as evidence-based demands which policy makers wish to supply [19], while demands in general mean the user’s expressed desire for a service or good that can be received for free or by purchase [20]. There have been arguments towards the needs-demands distinction, as in theory the concept of demands covers the idea of needs and can apply to public services [21]. As a result, the two terms of “needs” and “demands” have been often used interchangeably, which was taken in this study. So far, older residents in rural China can normally afford to buy food, drink and cloth for physical survival with the help of subsidies and supplements. They have proceeded to consider their safety, health and other needs. Zhang [22] found that older villagers were in greater demands for daily health care and medical services because of poor education and physical mobility. Zhong et al. [23] highlighted that there has been general ignorance of older people’s psychological needs from family members and the whole society. Further in Kong et al.’s study [24], older people expressed a very strong desire for interpersonal communication and constant companion, achieving 94.4 and 94.3% respectively. In addition, Niu et al.’s study [25] showed that older people in rural villages were more vulnerable to loneliness and social isolation than those in urban cities. Such loneliness and isolation are linked to higher risks of experiencing physical and mental problems that need to be addressed by more respect and deference. Meanwhile, older villagers especially those living alone or with a spouse were more likely to participate in social activities, such as village elections and economic development, which were positively associated with health-related quality of life [26].

After identifying the diversity of older people’s demands, empirical studies proceeded to investigate their priorities for the provision of related social services. According to Li’s research findings [27], there were mainly three types of demands in rural Henan province, including life care, medical care and mental health; among these, life care was the most urgent need, followed by medical care and mental health. In Zhang’s study, rural older people in Jiangsu province gave the first priority to medical services, next to entertainment services and domestic services [22]. Evidence from Wang’s study showed that Gansu older villagers exhibited the strongest preference for medical care and mortal care compared with life care and entertainment services [28]. Indeed, demands for various community care services among older people are likely to differ across different environmental and cultural settings. Some studies have explored factors that exerted an influence on demands for social care services in specific communities [29–31]. These factors have been classified into three categories [22]: (i) predisposing factors, reflecting differences in the demographic structure (e.g. age, education level) that driver the needs for care and support; (ii) risk factors, regarding inequalities in health (e.g. health status, physical function) that associate with the capability of ageing at home; (iii) enabling factors, concerning the availability of social and material resources (e.g. income, family resources) that affects the demands for help and services. This conceptual framework is often used to measure demands of older people for various types of community care services.

Despite the increasing diversity in service demands, the role of the family as the focus of care for older people has been modified by the rapid increase of labour migrations from rural to urban areas combined with the general emergence of the “4–2-1” family structure under the strict one-child policy [32]. Instead, there has been increased attention paid to the improvement of community-based supports and services for rural older people [33]. Until 2017, a total of 125,000 pension facilities had been built across rural China, including 43,000 social service agencies and 82,000 mutual aid organizations, constituting 59.4% of all rural communities [34, 35]. However, the overall level of service provision for older villagers remained relatively low, which might be affected by supply situation and service quality. Wang and Peng statistically analysed quality indicators of social care services and found that developed regions had the potential to deliver better services for older people ageing in rural communities [36]. Besides, social care services focused more on older people’s physical demands than on psychological health [37]. As investigated in Li’s research, owing to the influence of traditional Chinese culture, 73.4% of older people spent their leisure time on watching TV or chatting with neighbors, and 44% communicated less often with their children on difficulties and preferences [38]. On the whole, the supply of social care services for older people varied from one rural community to another, reflecting economic condition, social capacity and family support.

This study is aimed at investigating the current status of older people’s demands for social care services in rural communities and assessing the effectiveness of service supply. It seeks to address the following research questions: (i) what are the current demands of older people for healthy ageing in rural areas? (ii) which services have been provided by rural communities and to what extent have they been utilised? (iii) whether has the service supply satisfied the varying demands?

### Objectives of community care policies for older villagers

National government has issued a range of policies to improve the efficiency of the healthcare system and to increase the supply of low-cost and wide-ranging care for older persons. In 2008, the China National Committee on Ageing and other nine departments jointly published *Opinions on Comprehensively Promoting Home-based Care Services for Older People*. This policy aims to upgrade the service capability by developing home- and community-based care services in compensation for weakened traditional family support [39]. In 2017, the *Strategic Plan for Rural Revitalization* (2018–2022) proposed to develop the multi-layered old-age care system, which was based on the home, supported by the community and supplemented by the institutions, with the focus on improving the capability of rural healthcare facilities for the delivery of medical care and health services [40]. Meanwhile, *Opinions on Strengthening Care and Services for Left Behind Older People in Rural Areas* was issued to expand investments in welfare institutions and facilities, contributing to make basic care services more accessible to older people in rural villages [41]. Further in 2019, *Opinions on Promoting the Development of Care Services for Older People* was published to promote the integration of social care and medical services in rural communities as well as the establishment of mutual aid organizations for older people’s care such as rural mutual aid happiness home [42]. Apparently, expanding the concept of socialisation to community care and making good use of services from the family, community and institution are crucial to meet the rising needs of older people in rural areas.

In order to achieve national outcomes towards rural healthy ageing, local governments published their own policies and action plans for the delivery of community care services [43]. For example, in 2016, Department of Civil Affairs of Guangdong Province issued *Opinions on Happiness Plan Project to Improve Rural Care Services* to provide a wide range of social care for rural seniors with none or little cost, such as entertainment services, daily care and so on [44]. In 2018, Shanghai Civil Affairs Bureau and other eight departments.

Jointly issued a *Three-Year Action Plan for Better Life of Older People in Rural Areas (2018–2020)* to optimise the community care system for older people in rural villages [45]. In 2019, Department of Civil Affairs along with other three departments in Shanxi Province published *Action Plan for the Development of Rural Community Care (2019–2021)* to implement 11 projects for overcoming barriers to older people’s care in rural communities [46].

In Zhejiang province, the policy framework that sets out objectives to improve access to rural social care options in local communities mainly consists of four pieces of policies (Table 1). While the *Three-year Plan for the Development of Home-based Care Service Facilities for Older People* focuses on developing home-based community care facilities and expanding functions of community care services across rural villages, the *Implementation Opinions on Promoting the Development of Older People’s Care Industry* pays attention to the diversity of community care services and its financial resources. In response to the disparity of service quality between rural and urban areas, the *Implementation Opinions on Optimising Older People’s Care System and Improving Service Quality* highlights the construction of demonstrated residential care homes and the integration of different funding resources for the improvements in effectiveness and accessibility of care services in rural communities. After that, the *14th Five-year Plan for the Development of Older People’s Care Services* gives priority to the development of different community care facilities and services, such as residential care homes, mutual support, catering services and so on, in order to meet the increasing demands of older people in rural villages.

**Table 1:** Policy framework for the provision of community care services for rural older people

Policies	Policymakers	Objectives
Three-year Plan for the Development of Rural Home-based Care Service Facilities for Older People 2013	Department of Civil Affairs of Zhejiang Province	• To incorporate rural home-based care services into the social care service system; • To develop home-based social care facilities covering one third of rural communities by 2015; • To expand functions of home-based social care services, including information registration, demand assessment, daily life care, catering service, rehabilitation nursing, entertainment services and other voluntary services.
Implementation Opinions on Promoting the Development of Older People’s Care Industry 2014	The People’s Government of Zhejiang Province	• To increase public purchase funds and expand the supply of diversified and multi-level care services for older people; • To achieve full coverage of home-based community care service centres in rural areas by 2017; • To encourage rural old age association to participate in management of the care service centre; • To guide private sectors to invest in residential care homes in rural villages.
Implementation Opinions on Optimising Older People’s Care System and Improving Service Quality 2018	The People’s Government of Zhejiang Province	• To promote the construction of rural demonstrated residential care homes; • To improve care services for older people with disabilities and dementia; • To increase finance support for the construction of rural care facilities and residential care homes.
The 14th Five-year Plan for the Development of Older People’s Care Services 2021	Department of Civil Affairs of Zhejiang Province	• To develop community catering services and domestic assistance for older people; • To improve residential care homes for rural older people; • To promote mutual support in old care across villages; • To increase funds for the supply of basic care services for older people in rural areas.

## Materials and methods

### Research strategy

This study adopts a quantitative approach to investigate the demands of older people and the supply of community care services in rural Zhejiang province. It chose to focus on Zhejiang rural villages, there are two reasons for this. First, Zhejiang has one of the fastest-growing ageing population in China. Until 2019, older population aged 60 and over were around 12 million, taking up 23% of the total population in the province, and is projected to reach almost 16 million (25%) in 2025. Second, Zhejiang province has a highly diversified economy, along with many cities and counties contributing a heavy investment to rural development and social welfare for older people.

In 2018, Department of Civil Affairs of Zhejiang Province published *Notification of Assessment on Rural Community Construction for Demonstration* to modernise rural communities and innovate care services. This notification sets a clear goal and achievement strategy to promote socio-economic development of all rural communities and turn into demonstration sites. Villages were self-recommended to be assessed and selected as demonstration sites. As a results of assessments, 304 villages were selected in the first instance of 2018, which was followed by 376 villages on the second selection in 2019. A questionnaire survey was carried out at the village level to understand their older people’s overall demand for and supply of care services in each rural community; sample was drawn from these demonstration villages. The rationale for this choice is threefold. Firstly, demonstration villages have seen considerably economic success and paid increasing attention to the provision of welfare facilities and healthcare services. Secondly, all rural villages are in need of upgrading their community facilities and services to meet the selection criteria for demonstrations. Examining the demonstrations can offer policy implications of establishing advanced social care practices in developed as well as underdeveloped rural communities. Thirdly, older residences in rural demonstration areas have relatively greater income and higher expectation for healthy ageing, which will be a universal tendency across other rural villages.

### Data collection

Following a systematic review of policy reports and journal articles, a questionnaire survey was designed and divided into three parts, covering the demands of older people, the supply of community care services, the utilisation and preferences for community care services at the village level. As explained above, predisposing (e.g. demographic structure), risks (e.g. health status) and enabling (e.g. socioeconomic resources) are usually three types of factors that can influence the demands of older people for community care services [22]. This conceptual framework has been adopted to measure service demands among older people. Accordingly, certain questions were included in the questionnaire survey to investigate details and statistics on demographic features (e.g. age/sex distributions of the older population, levels of educational attainment), health status (e.g. living arrangements of older persons, current status of functional capacity) and socioeconomic characteristics (e.g. average monthly income and expenditure among older people, total annual village collective revenues). In addition, based on two key determinants of supply situation and service quality, combined with the objectives of rural healthy ageing policies, questions related to the categories of community care services, the construction of residential care homes and the provision of catering services have been developed for measurement of community care service provision. Since ranking public services can provide an insight to the comparative assessment of service performance and the strengths or weakness of service attributes [47], a group of general community care services have been listed for rankings in the descending order from the most to the least frequently used services. Also, the ranking technology has been used to find out older villagers’ preferences for community care services, ranging from 1 (the most preferred service) to 5 (the least preferred service).

Prior to the main survey, a pilot test was conducted with 56 village communities to ensure the clarity and brevity of all the questions, which resulted in modification of some questions. Once the questionnaire was finalized, it was sent out with the cover letter by traditional mail and by online system to the community centre in 207 rural villages across 9 cities, including HZ1, HZ2, JX, JH, LS, NB, CZ, SX and WZ. Reminder phone calls were made to non-respondents after 2 weeks. The survey was generally answered by the village representative who is responsible for the provision and management of community care services, mainly on the basis of daily records of service or facility use. Take community canteen services for instance, the village committee first has the registration of older persons in demand for catering services and then ticks their names when they come to the canteen for lunch or dinner. For outcome monitoring and performance improvement, some villages often carried out an assessment of their community care services. Consequently, these service performance feedback and service management observations were used as supplements for the assessments of utilisation and preferences for social care services in rural communities. In the end, a total of 186 valid responses were received, with a response rate of 89.9%. Table 2 showed a variety of response rates from 67.9 to 100% between different cities. It is difficult to generalise the reasons for those who did not reply, apart from village officials’ indifference to academic research. A post-survey analysis found that non-respondents shared characteristics of having limited access to collective revenue or community care services.

**Table 2:** Descriptive statistics of survey responses

Cities	Number of questionnaire sent out	Number of valid responses received	Response rate
HZ1	28	19	67.9%
HZ2	19	17	89.5%
JX	36	36	100%
JH	15	15	100%
LS	16	15	93.8%
NB	18	18	100%
CZ	29	27	93.1%
SX	15	15	100%
WZ	31	24	77.4%
Total	207	186	89.9%

### Data analysis

Survey data were coded and analysed by using the Statistical Package for the Social Science (SPSS) [48]. Frequency tables displayed demonstration villages’ responses to separate variables in each question, which measured similarities and differences in the demand and supply of social care services among rural communities. Comparative analysis examined the balance between service demand and service supply. Bivariate analysis was conducted to identify relationships between variables and to determine key factors that have impacted the provision of social services.

## Results

### Demands of older villagers for community care services

Table 3 disclosed the demands of older population for community care services in rural areas from three aspects of demographic structure, health status and socioeconomic characteristics. In terms of demographic structure, 105 of 186 rural communities had a population size of less than 2001, only 21 villages had a greater population density than 4000. The 60–69 age group accounted for more than half (53.6%) of the total older population, while the age group 70–79 shared another 30.3%. By comparison, older women (51.1%) slightly outnumbered their male counterparts (48.9%), 69.6% of older villagers have received education at the primary school or lower, in contrast with just 2.8% holding college or higher degrees.

**Table 3:** Descriptive statistics of variables regarding demands of older people for community care services

Survey questions	Yes (n)	%
*Demographic structure*		
Total number of population in the village		
1000 and below	29	15.6%
Between 1001 and 2000	76	40.9%
Between 2001 and 3000	32	17.2%
Between 3001 and 4000	28	15.1%
4001 and above	21	11.3%
Distribution of older people aged 60 and over		
Between 60 and 69	54,441	53.6%
Between 70 and 79	30,730	30.3%
Between 80 and 89	14,230	14.0%
90 and above	2183	2.1%
Number of males and females aged 60 and over		
Male	59,891	48.9%
Female	62,703	51.1%
Levels of educational attainment		
College and higher	3086	2.8%
High school	6697	6.1%
Middle school	23,524	21.5%
Primary school and lower	76,156	69.6%
*Health status*		
Living arrangements		
Live alone	4753	4.9%
Live with spouse	34,919	34.4%
Live with children	59,342	58.4%
Other	2570	2.5%
Older people have their own property		
Yes	52,657	27.6%
No	137,839	72.4%
Status of functional capacity		
Able to perform activities of daily living completely	77,115	77.0%
Able to perform activities of daily living basically	18,921	18.9%
Dependent on others basically	2769	2.7%
Dependent on other completely	1367	1.4%
Status of primary care support		
Care by self	125	40.5%
Care by spouse	84	27.2%
Care by children	91	29.4%
Other	9	2.9%
*Socioeconomic characteristics*		
Level of village collective revenue (CNY)		
500 k and below	82	44.1%
Between 510 k and 1000 k	26	14.0%
Between 1001 k and 1500 k	19	10.2%
1501 k and above	59	31.7%
Per capital disposable income in the village (CNY)		
10,000 and below	28	16.4%
Between 10,001 and 20,000	39	22.8%
Between 20,001 and 30,000	40	23.4%
Between 30,001 and 40,000	44	25.7%
40,001 and above	20	11.7%
Age pension paid monthly by the village (CNY)		
None	17	10.1%
Between 1 and 500	133	79.2%
Between 501 and 1000	16	9.5%
Between 1001 and 1500	0	0.0%
1501 and above	2	1.2%
Level of monthly income (CNY)		
500 and below	33	17.7%
Between 501 and 1000	76	40.9%
Between 1001 and 1500	59	31.7%
1501 and above	18	9.7%
Level of monthly spending (CNY)		
500 and below	47	25.3%
Between 501 and 1000	111	59.7%
Between 1001 and 1500	26	14.0%
1501 and above	2	1.1%

From the perspective of health status, 92.8% of older people lived with either their children (58.4%) or their spouse (34.4%), almost three quarters of older people did not have their own homes (72.4%). 77.0% of older population were able to carry out daily life activities independently and effectively, compared with 1.4% being fully dependent on others. Accordingly, older people in 40.5% of rural communities were mainly cared by themselves, while in the remaining villages core care and support were delivered by the spouse (27.2%) or children (29.4%).

With respect to socioeconomic characteristics, the level of annual village collective revenue varied significantly from 78 k Chinese Yuan Renminbi (CNY) to 35.3 m CNY. 82 of 186 villages obtained less than 500 k CNY of the collective revenue, compared with 59 villages receiving the collective income over 1500 k CNY. There was a wide variety of the average available money per person after income taxes across 123 villages, ranging from 10 k CNY to 40 k CNY. 89.3% of villages provided their older adults with less than 501 CNY as monthly pension, including 10.1% of them being unable to pay any age pension. Of the 186 rural villages, the median monthly income for older people in 72.6% was between 501 CNY and 1500 CNY, compared with that in 17.7% being less than 500 CNY and in 9.7% being more than 1500 CNY. Correspondingly, on average older people spent less than 1000 CNY every month in 85.0% of rural communities, only in 2 villages the average monthly expenditure was higher than 1500 CNY.

Overall, village collective revenue plays an essential role in addressing service demands among older people seeking to age at home. Table 4 showed that the level of village collective revenue had a positive impact on the payment of old age pension, older people’s monthly income and the personal disposable income. Villages with an annual collective revenue of over 1500 k CNY tended to have a per capital disposable income of 30 k CNY or above, while those, who received a revenue collection of 500 k CNY or less, often had the per capital disposable income of 20 k CNY or below. There were wide differences in the benefit of old age pension, villages, who reported to have higher collective earnings, appeared to provide extra pension money for their older people. Meanwhile, of those older people who earned a regular income of over 1500 CNY or had after-tax income of over 40 k CNY, 50% lived in rural villages with the collective revenue of over 1500 k CNY.

**Table 4:** Crosstabs between village collective revenue and disposable income, monthly pension and monthly income

*Level of village collective revenue*	Per capital disposable income in the village (CNY)				
	10,000 and below	Between 10,001 and 20,000	Between 20,001 and 30,000	Between 30,001 and 40,000	40,001 and above
500 k and below	14 (50.0%)	24 (61.5%)	17 (42.5%)	13 (29.5%)	5 (25.0%)
Between 510 k and 1000 k	4 (14.3%)	6 (15.4%)	5 (12.5%)	7 (15.9%)	1 (5.0%)
Between 1001 k and 1500 k	2 (7.1%)	2 (5.1%)	4 (10.0%)	7 (15.9%)	4 (20.0%)
1501 k and above	8 (28.6%)	7 (17.9%)	14 (35.0%)	17 (38.6%)	10 (50.0%)
Total	28 (100%)	39 (100%)	40 (100%)	44 (100%)	20 (100%)
	Age pension paid monthly by the village (CNY)				
	500 and below	Between 501 and 1000	Between 1001 and 1500	1501 and above	
500 k and below	66 (44.0%)	1 (6.3%)	0 (0.0%)	1 (50.0%)	
Between 510 k and 1000 k	22 (4.7%)	2 (12.5%)	0 (0.0%)	0 (0.0%)	
Between 1001 k and 1500 k	19 (12.7%)	0 (0.0%)	0 (0.0%)	0 (0.0%)	
1501 k and above	43 (28.7%)	13 (81.3%)	0 (0.0%)	1 (50.0%)	
Total	150 (100%)	16 (100%)	0 (0.0%)	2 (100%)	
	Level of monthly income (CNY)				
	500 and below	Between 501 and 1000	Between 1001 and 1500	1501 and above	
500 k and below	21 (63.6%)	32 (42.1%)	23 (39.0%)	6 (33.3%)	
Between 510 k and 1000 k	1 (3.0%)	18 (23.7%)	7 (11.9%)	0 (0.0%)	
Between 1001 k and 1500 k	5 (15.2%)	7 (9.2%)	4 (6.8%)	3 (16.7%)	
1501 k and above	6 (18.2%)	19 (25.0%)	25 (42.4%)	9 (50.0%)	
Total	33 (100%)	76 (100%)	59 (100%)	18 (100%)	

### Supply of community care services in village communities

Following the buildup of social and economic capacity, some rural communities have been in a position to provide a wider range of social welfare benefits and social care services [49]. Service provision and quality outcomes were reported to be two key determinants of community care supply. Table 5 describes statistic results associated with the supply of community care services for older villagers across rural Zhejiang province. Entertainment services (ES) have been given the prevalence, 88.8% of village communities have designed the entertainment platform for their older people. It was quite common to see the provision of daily life care (DLC, 74.2%) and legal aid (LA, 72.5%) among rural areas, while there were a relatively small number of villages offering domestic services (DS, 41.0%) and rehabilitation nursing (RN, 49.4%). There was a correlation between the level of village collective revenue and the supply of community care services (Table 6). Villages, who reported an annual income of over 1500 k CNY, appeared to provide four or more categories of community care services, while those, who received the village collective revenue of 500 k CNY and below, tended to have fewer than three service types.

**Table 5:** Descriptive statistics associated with the supply of community care services for older people

Supply of community care services	Yes (n)	%
Daily life care	132	74.2%
Domestic services	88	49.4%
Rehabilitation nursing	73	41.0%
Medical care	123	69.1%
Psychological comfort	103	57.9%
Entertainment services	158	88.8%
Legal aid	129	72.5%
Types of residential care home		
Yes	133	72.7%
Public	83	62.4%
Public-to-private	31	23.3%
Private	12	9.0%
Other	7	5.3%
No	50	27.3%
Provision of community canteen for older people		
Yes	135	72.6%
With good quality	5	3.7%
With normal quality	49	36.3%
With poor quality	81	60.0%
No	51	27.4%

**Table 6:** Crosstabs between village collective revenue and service provision

*Level of village collective revenue*	Categories of services (n/%)							
	0	1	2	3	4	5	6	7
500 k and below	6 (75.0%)	6 (66.7%)	8 (44.4%)	18 (58.1%)	10 (31.3%)	14 (51.9%)	7 (31.8%)	13 (33.3%)
Between 510 k and 1000 k	0 (0.0%)	2 (22.2%)	3 (16.7%)	4 (12.9%)	7 (21.9%)	3 (11.1%)	3 (13.6%)	4 (10.3%)
Between 1001 k and 1500 k	0 (0.0%)	0 (0.0%)	4 (22.2%)	2 (6.5%)	5 (15.6%)	0 (0.0%)	3 (13.6%)	5 (12.8%)
1501 k and above	2 (25.0%)	1 (11.1%)	3 (16.7%)	7 (22.6%)	10 (31.3%)	10 (37.0%)	9 (40.9%)	17 (43.6%)
Total	8 (100%)	9 (100%)	18 (100%)	3 (100%)	32 (100%)	27 (100%)	22 (100%)	39 (100%)

Residential care home (RCH) has been increasingly recognized as an important support for older people, especially for those with disabilities or chronic illnesses [50]. 72.7% of rural villages have developed various types of RCHs, including public residential care homes (45.3%), publicly constructed and privately-run residential care home (16.9%), private residential care homes (6.6%) and others (3.8%). However, there were still 27.3% of village communities short of RCHs. Community canteen services (CCS) for older adults were rather universal, with 72.6% of villages offering this service. However, there were certain worries about the quality of CCS, as 70.3% of respondents considering such services as poor or normal.

### Utilisation of community care services by older people

**Fig. 1:**
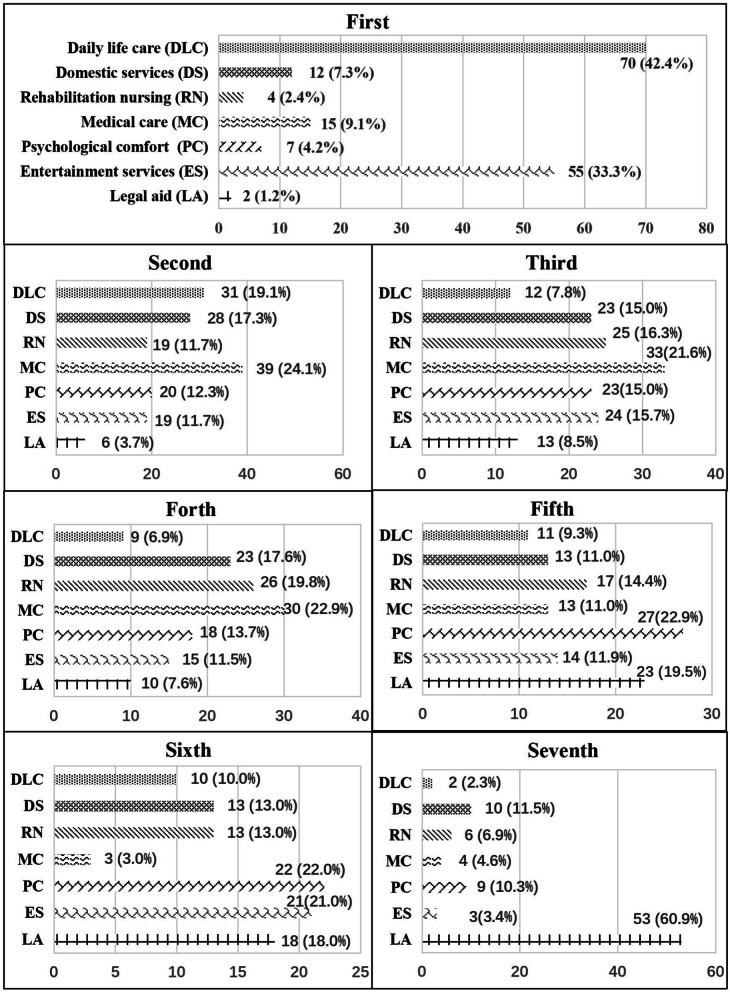
The ranks of utilisation of community care services by older people

Although a variety of community care services are available to older people in each village, their utilisation differs across Zhejiang Province. Figure 1 showed the rank orders of 7 community care services from the most (1) to the least utilisations (7) by older people in rural villages. In the first ranking, older people in a majority of rural villages were likely to use DLC (42.4%) and ES (33.3%). Medical care (MC) and DLC have been ranked as the second most popular services by a relatively large number of villages, 24.1 and 19.1% respectively, which was followed by DS among 17.3% of rural areas. Interestingly in the third ranking, different villages have given priorities to different types of community care services, resulting in almost identical utilisation of RN (16.3%), ES (15.7%), DS (15.0%) and psychological comfort (PC, 15.0%). MC continued to be recongnised as the most utilised service by 21.6 and 22.9% of rural villages in the third and fourth rankings. From the ranking of fifth, PC and LA took up the largest proportions, with the least utilisation of LA being ranked seventh by 60.9% of rural villages.

**Fig. 2:**
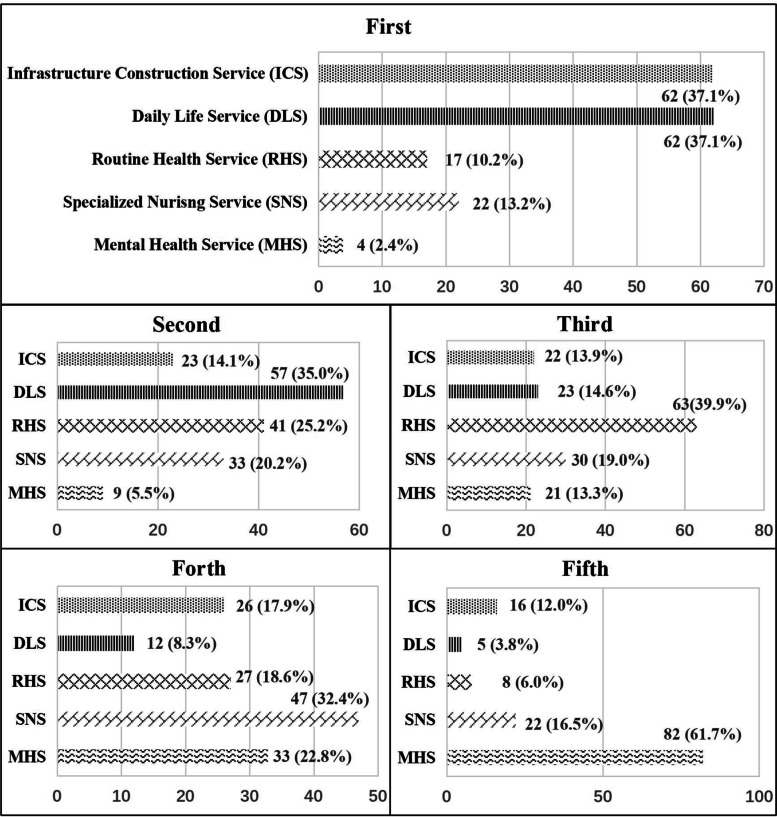
The ranks of rural preference for community care services

Economic growth and demographic change are creating diversity in the demands of older villagers for community care and in the capability of village communities to provide services for older people. Figure 2 displays the rank orders of 5 community care services from the most (1) to the least (5) preferences among older people in rural areas. Both infrastructure construction service (ICS) and daily life service (DLS) were ranked as the most desirable services for older adults by 37.1% of rural villages, while there were only 2.4% of rural villages placed the greatest value on mental health service (MHS). DLS continued to receive the most support of 35.0% from 57 rural villages in the second ranking, compared with 5.5% giving priority to MHS. At the third and fourth rankings, there was a clear preference for the routine health service (RHS) and specialized nursing service (SNS), 39.9 and 32.4% respectively. MHS was viewed as the least important service, with the majority of rural villages (61.7%) choosing it in the last ranking.

## Discussion

There is a clear diversity in the demands for community care services among older people in rural areas on the basis of demographic structure, health status and socioeconomic characteristics. The number of population varied greatly across rural villages from less than 1000 to over 4000, with most older people aged between 60 and 79. This indicates an increasing number of the oldest-old that will lead to greater needs for care and support in local communities. According to Kubzansky et al.’s study [51], the lower levels of education are often linked with the poorer psychological, behavioral and biological conditions among older people that could influence older people’s health and increase their demands for social care. Currently, educational levels in rural villages were relatively low, with over two thirds of older residents having just completed primary school or been illiterate. Indeed, these older people will need more social support and care services as they grow older.

Living arrangements and health status play a large role in predicting the demands for community care service. At present, older villagers frequently live with either their spouse or children, few of them have their own property. This implies that the tradition of several generations living together under the same property remains prevalent in the rural society. Because of the accessibility to family support, older people might be less likely to seek for community care services [52]. In addition, most older people are generally able to carry out activities of daily living, they tend to take care of themselves and maintain their independence. Despite this, in more than half of rural communities older population relied heavily upon their spouses or children. Such demographic pressures present numerous challenges to the family as the main source of care for older people, especially when labor migration from rural areas keeps expanding [53].

Rural communities have been imbued with positive functionalities of providing social services and place attachment [54]. In China, there have been an increasingly differentiated picture of economic development and social capacity among village communities [49, 55]. Such differences can greatly impact upon older people’s ability to age at home. The survey found that, the collective income available to rural communities varied markedly, leading to a wide gap between the richest and the poorest villages. Rural villages, who claimed to obtain a higher level of collective revenue, granted a larger pension supplement to their older members. Accordingly, older residents living in the more prosperous rural areas tended to have a greater level of monthly income and disposable income. However, economic status was found to be insufficient as a whole, with most villages reporting older residents’ income and spending of less than 1000 CNY per month. This contradicts the expected increase in consumer expenditure as price rises, which can affect both affordability and acceptability of community care services.

To address these challenges, national policies (e.g. *Opinions on Comprehensively Promoting Home-based Community Care Services for Ageing*) were published to improve access to and the quality of community care services as well as to reduce unmet needs caused by shrinking family size and social bonds. The survey found that a broad range of social services have been delivered to meet the varying needs among older residents across rural communities. Of these, there were very generous provision of ES and DLC. This corresponds to other studies, such as Hossain et al. [56] and Yuan [57], which reported that entertainment support and daily care can contribute to the overall well-being and health of older people. In contrast, comparatively less attention has been given to DS and RN, meaning that some of safety needs are often devalued. This is probably because economic resources in rural communities were insufficient for the delivery of different social care services. In fact, the level of village collective revenue had a positive influence upon the supply of community care services. To be specific, the more collective income a village had, the wider social services provided by the community.

RCH is crucial to meet the support needs of older people with disabilities or dementia [58]. However, nearly one third of rural villages were still lack of RCHs, and few of RCHs were organised and financed by private enterprises. There needs to be better policy towards greater advocacy for the participation of private sectors in the construction and development of rural care services for older people. Older people, especially those living alone, always found independent cooking as a burdensome [59], CCSs can positively impact their mental health and life satisfaction to support successful ageing in place [60]. Unfortunately, a certain number of rural villages did not establish any canteens for their older members. One possible explanation is that financial support for some essential services has not been prioritised and guaranteed in such rural communities [61]. There were further concerns about the quality of CCSs, because 60% of rural communities described their canteen services as “poor” and only 5 villages gave the “good” answer. Therefore, it is essential that local government sets guidelines to improve the supply and the efficiency of community care services in rural areas.

Meeting older people’s demands for community care services has been recognised as the key benchmark against which a better life is guaranteed [62]. Although some rural communities have been in a position to provide a variety of social care services, the utilisation of these services remained relatively limited. According to the survey, DLC and ES were used most by older population in rural areas, followed by MC, DS and RN. By comparison with the high supply of LA and PC, their utilisation rates were substantially low with most villages ranking them the sixth and seventh. These demands might be met by family support or social network to some extent. However, there have been an observed mismatch between the demand for and the supply of community care services, leading to inevitable waste of public resources. To address this mismatch and support older villagers healthy ageing, it is necessary to understand the potential demand for social care services as well as to improve the service diversity within rural communities, including ICS, DLS, RHS, SNS and MHS [52].

Construction of care service infrastructure plays an important part in maintaining functional independence in old age [63]. As evidenced by the survey findings, there were the strongest preference for ICS and DLS among older people in the rural society. By contrast, little attention has been paid to MHS and SNS. One inference from this could be that older villagers used to ignore their feelings of belongingness and dignity [64]. Besides, financial affordability might be in position to influence personal preference [65]. According to the Maslow’s hierarchy of needs [66], there are five levels of human needs including physiological needs, safety needs, belongingness and love needs, esteem needs and self-actualization, every person has the desire to move up from the bottom level of physiological and safety needs toward the top level of self-actualization. Ironically, progress in rural villages is rather pessimistic with most of older residents focusing on the achievement of lower level deficit needs.

### Limitations

While this study brought fresh insights into current demand and supply of community care services for older people in rural areas, there are some limitations. First, this study investigated service demand and supply among older population at the village level instead of the individual level, the empirical results need to be carefully explained. It focused on more prosperous villages in Zhejiang province, there were the differentiation of socio-economic capacity of rural communities to support healthy ageing, the research findings might not be generalisable for underdeveloped rural areas. Further actions are needed to extend the research work towards other rural villages. Besides, due to the limited records of service performance, observation was also used to assess the utlisation and preference of community care services, there might be bias and judgments towards the survey questions, which was recognised by others [67]. Meanwhile, key informants might have a conflict of interest in the role of village representatives, cautions should be paid. Another difficulty was caused by variations in community care service provision for older people, not all questions were applicable to all rural villages.

## Conclusion

Understanding older people’s demands for community care services plays a crucial role in enhancing the efficiency of service provision and supporting older people healthy ageing in place. There have been increasingly diversified demands for care and support among older people across village communities. National and local governments have placed a particular emphasis on the growth in supply of community care services within rural villages. However, the study found that the categories of care services provided by rural communities remained relatively small compared with the diversity of service demands brought by economic development and demographic change.

Community prosperity is able to expand social benefits and enhance formal support for older residents. However, the economic and social capacity of rural communities varied significantly, resulting in a distinct picture of service provision across Zhejiang province. It seems inevitable that limited social capital and accessible resources will place a heavy reliance on family and relatives. Expanding capital investments towards socially necessary services can improve the accessibility of community care resources for older villagers to age in their familiar environment.

All in all, ES and DLC were in common supply, while less attention have been paid to DS and RN. Despite the importance of RCH for vulnerable older people, it was unavailable in some rural villages and still mainly funded by local government. Policy advocacy is needed to improve private sectors participation in the development of social care services in rural community. Although many rural communities have offered canteen services for their older members, there should be guidelines in place to guarantee service quality. Except DLC and ES, other services have been used to a limited extent. There were an observed mismatch between the demand and supply of social services across rural communities. Such mismatch could be compensated by an in-depth understanding of the potential demands as well as an effective provision of community care services. There have been general expectations and perceptions of both ICS and DLS, while MHS and SNS continues to be ignored by older population in most rural villages. A range of positive actions should be taken to satisfy basic needs and higher order needs.

## Data Availability

Quantitative data generated and analysed during the research are not available publicly to maintain confidentiality, but can be available on reasonable request.

## Abbreviations

CCS: Community canteen services
CNY: Chinese Yuan;
DLC: Daily life care
DLS: Daily life service
DS: Domestic services
ES: Entertainment services
ICS: Infrastructure construction service
LA: Legal aid
N: Number
MC: Medical care
MHS: Mental health service
PC: Psychological comfort
RCH: Residential care home
RHS: Routine health service
RN: Rehabilitation nursing
SNS: Specialized nursing service
